# Murine leukemia viruses (MuLV) and Xenotropic MuLV-related viruses exhibit inter-tropic complex recombination patterns

**DOI:** 10.1186/1742-4690-8-S1-A235

**Published:** 2011-06-06

**Authors:** Mattia C F Prosperi, William M Switzer, Walid Heneine, Marco Salemi

**Affiliations:** 1 Department of Pathology, Immunology and Laboratory Medicine, Emerging Pathogens Institute, College of Medicine, University of Florida, Gainesville, Florida, USA; 2 Laboratory Branch, Division of HIV/AIDS Prevention, National Center for HIV/AIDS, Viral Hepatitis, STD, and TB Prevention, Centers for Disease Control and Prevention, Atlanta, GA, 30333, USA

## Background

Murine leukemia viruses (MuLV) are classified into three groups by host tropism correlating with viral receptor sequences present in the surface protein of env. Ecotropic MuLV are found only in mice, xenotropic replicate in non-mouse cells, and polytropic in mouse and non-mouse hosts. Xenotropic MuLV-related viruses (XMRV) and polytropic MuLV have been found in humans and reported in different diseases, including prostate cancer and chronic fatigue syndrome. Classification of MuLV tropism is typically done using small portions of gag/pol, however, this may be complicated by viral recombination.

## Methods and results

Eight eco/poly/xenotropic MulV complete genomes were selected from GenBank, plus an XMRV sequence. The dataset showed strong phylogenetic signal. By tropism-group analysis using SimPlot software, XMRV was detected as a mosaic recombinant form of all three groups. Using more sensitive analyses, we found evidence of inter-tropic recombination, especially outside of env. Different procedures for recombination analysis (SimPlot/TOPALi2), applied to the whole dataset independently from the tropism, inferred discordant break-points.

## Conclusions

Given the evidence of inter-tropic recombination in MuLV, detection and classification of recombination in XMRV using different MuLV tropism prototypes should be interpreted with caution. Despite using a small dataset, a strong phylogenetic signal in the alignments and highly resolved phylogenies inferred both by full-length and sliding-window approaches, different recombination programs reported conflicting results. These results suggest that identification of parental strains of the potential recombinants is difficult and that recombination in the highly genetically related MuLV have been occurring for some time.

